# Dihydromyricetin promotes autophagy and attenuates renal interstitial fibrosis by regulating miR-155-5p/PTEN signaling in diabetic nephropathy

**DOI:** 10.17305/bjbms.2019.4410

**Published:** 2020-08

**Authors:** Liming Guo, Kuibi Tan, Qun Luo, Xu Bai

**Affiliations:** Department of Nephrology, HwaMei Hospital, University of Chinese Academy of Sciences, Zhejiang, China

**Keywords:** Dihydromyricetin, DHM, autophagy, renal tubulointerstitial fibrosis, miR-155-5p, PTEN, PI3K, AKT, mTOR, diabetic nephropathy, NRK-52E cells

## Abstract

Diabetic nephropathy (DN) is the most common complication of diabetes and is prone to kidney failure. Dihydromyricetin (DHM) has been reported to have a variety of pharmacological activities. This study aims to explore the effect of DHM on DN and the underlying molecular mechanism. An *in vivo* DN rat model was established. The degree of renal interstitial fibrosis (RIF) was detected by hematoxylin-eosin (HE) staining, Masson’s trichrome staining, and immunohistochemistry (IHC). *In vitro*, NRK-52E cells were divided into four groups: normal glucose (NG), high glucose (HG), HG+DHM, and HG+rapamycin (autophagy inhibitor). The levels of autophagy- and fibrosis-related proteins were analyzed by western blotting. The expression of miR-155-5p and phosphatase and tensin homolog deleted on chromosome ten (PTEN) and their relationship were assessed by quantitative reverse transcription (qRT)-PCR and dual luciferase reporter gene assay. Our results showed that RIF was increased in DN rat model and in HG-induced NRK-52E cells. DHM treatment attenuated the increased RIF and also increased autophagy. MiR-155-5p expression was increased, while PTEN expression was decreased in DN rat and cell model, and DHM reversed both effects. Dual luciferase assay showed that PTEN was the target gene of miR-155-5p. DHM inhibited HG-induced fibrosis and promoted autophagy by inhibiting miR-155-5p expression in NRK-52E cells. In addition, DHM promoted autophagy by inhibiting the PI3K/AKT/mTOR signaling pathway. In conclusion, DHM promotes autophagy and attenuates RIF by regulating the miR-155-5p/PTEN signaling and PI3K/AKT/mTOR signaling pathway in DN.

## INTRODUCTION

Diabetes mellitus (DM) is a chronic systemic metabolic disease caused by absolute or relative deficiency in insulin secretion in the body [1]. Due to the increasing incidence of DM diabetic nephropathy (DN, a complication of DM) is gradually becoming one of the most common microvascular complications worldwide [2,3]. Previous studies found that DN is usually caused by the interaction of environmental and genetic factors [4]. Internal environment disorders and metabolic abnormalities caused by hyperglycemia are the factors that cause DN [4,5]. In addition, the proliferation of mesangial cells, enlargement of the mesangium, and accumulation of abnormal extracellular matrix (ECM) can lead to renal interstitial fibrosis (RIF), ultimately leading to chronic renal failure and promoting the occurrence of DN [5,6]. Therefore, new diagnostic strategies and therapeutic targets are urgently needed for proper diagnosis and treatment of DN.

Dihydromyricetin (DHM, CAS#: 27200-12-0) is a kind of flavonoids extracted from *Ampelopsis* Michx. [7] that has many pharmacological activities, including antioxidant, anti-inflammatory, antihypertensive, anticancer, free radical scavenging, and anti-fatigue activities [8]. Xu et al. found that DHM can delay the progression of DM and its complications, thus improving the life quality of patients [9]. DN may be caused by inhibition of autophagy of renal cells and abnormal deposition of ECM [10]. Inhibition of adenosine monophosphate-activated protein kinase (AMPK) activity in DN can inhibit the activation of mammalian target of rapamycin (mTOR) and promote cell autophagy to reduce the damage of kidneys [11]. Studies revealed that DHM promotes cell autophagy to improve skeletal muscle insulin sensitivity in nonalcoholic fatty liver disease (NAFLD) patients [7,12]. However, another study reported that DHM promotes autophagy and inhibits transforming growth factor-beta (TGF-β) expression by activating the AMPK/mTOR signaling pathway in DN [13]. DHM may play a protective role in early renal injury, which can prevent the occurrence and development of DN [14].

MicroRNAs (miRNAs) regulate the expression of target genes [15]. Previous studies showed that the expression level of miR-155 was downregulated in tissues and urine samples of DN patients [16,17]. The abnormal expression of miR-155-5p in DN patients may be related to the disease stage of patients or the pathogenesis of DN [17].

In this study, we established a DN rat model and NRK-52E cell model to investigate the effect of DHM on RIF and autophagy in DN and to explore the underlying molecular mechanism.

## MATERIALS AND METHODS

### Rat model

A total of 21 male specific-pathogen-free (SPF) Sprague-Dawley (SD) rats (6 weeks old), weighing 180 ± 20 g, were purchased from Jrdun Biotechnology (Shanghai, China) and randomly divided into three groups: negative control (NC) group, DN group, and DN+DHM group. A DN rat model was established according to the previous studies [18]. Briefly, the right kidneys of rats were excised in DN group and DN+DHM group, while the rats in NC group were subjected to sham surgery without renal damage, including laparotomy and renal pedicle surgery. One week after uninephrectomy, rats in DN and DN+DHM group were injected intraperitoneally with streptozotocin (STZ, 50 mg/kg, Sigma-Aldrich, St. Louis, USA) dissolved in 0.1 mM citrate buffer at pH 4.5, and NC group was injected with an equal volume of citrate buffer. Three days later, the blood glucose level of rats exceeded 16.6 mmol/L and the DN rat model was considered to be successfully established. Rats in DN+DHM group were treated with DHM (Xi’an Orient Biotechnology Co., Ltd, Xi’an, China) at a dose of 100 mg/kg/d for 10 weeks. The other groups were given the same volume of distilled water.

All animal experiments were approved by the Laboratory Animal Management Committee of HwaMei Hospital, University of Chinese Academy of Sciences for the use of animals and conducted in accordance with the National Institutes of Health (NIH) Laboratory Animal Care and usage guidelines (2019-245).

The rats were anesthetized with 10% chloral hydrate (30 mg/kg, Bright Chemical Co., Wuhan, China) intraperitoneally, the abdominal aorta of the rat was perfused with normal saline, and the kidney was washed *in situ*. Finally, the kidney tissues were taken out for subsequent experiments.

### Cell culture and transfection

Rat renal tubular epithelial NRK-52E cells and human embryonic kidney 293 (HEK293) cells were purchased from American Type Culture Collection (ATCC; Manassas, USA) and cultured in Dulbecco’s modified Eagle medium (DMEM, Sigma-Aldrich, St. Louis, USA) containing 5% calf bovine serum (CBS, Sigma-Aldrich, St. Louis, USA) and 0.05% dimethyl sulfoxide (DMSO, Sigma-Aldrich, St. Louis, USA) at 37°C.

NRK-52E cells were divided into four groups: normal glucose (NG) group, high glucose (HG) group, HG+DHM group, HG+LY294002 group and HG+rapamycin group. The cells in NG group were stimulated with 5.5 mmol/L glucose, in HG group with 30 mmol/L glucose, in HG+DHM group with 30 mmol/L glucose and 1 µM DHM, in HG+ LY294002 group with 30 mmol/L glucose and PI3K inhibitor LY294002 (MedChemExpress, New Jersey, USA), and in HG+rapamycin group were stimulated with 30 mmol/L glucose and the autophagy inhibitor rapamycin (Acmec Biochemical, Shanghai, China).

MiR-155-5p inhibitor, miR-155-5p mimic NC inhibitor, or NC mimic were transfected into NRK-52E cells at 37°C for 48 h using Lipofectamine 3000 (Thermo Fisher Scientific, Waltham, USA).

### The measurement of RIF degree

The degree of RIF was assessed by hematoxylin-eosin (HE), Masson’s trichrome staining, and immunohistochemistry (IHC). Kidney tissues were fixed with formaldehyde, embedded in paraffin, sliced, soaked in 40°C water, and then washed in phosphate buffered saline (PBS) three times. First, some paraffin tissue sections were stained with hematoxylin staining solution (Sigma-Aldrich, St. Louis, USA) for 10 min. A part of these sections was added eosin staining solution (Beyotime, Suzhou, China) for 3 min and dehydrated in ethanol. Other sections were stained with Masson bluing solution (Solarbio, Beijing, China) for 5 min, washed in distilled water, stained with Ponceau S staining solution (Solarbio, Beijing, China) for 8 min, re-washed in phosphomolybdic acid solution (Solarbio, Beijing, China), re-stained with aniline blue staining solution (Solarbio, Beijing, China) for 2 min, and dehydrated in ethanol. In addition, some tissue sections were blocked with 10% goat serum blocking solution (Solarbio, Beijing, China) for 35 min, respectively, added collagen-IV (Col IV) antibody (Yanjin Biological, Shanghai, China) and secondary antibody (Abcam, Cambridge, USA) to incubate at 37°C, washed twice in PBS (Solarbio, Beijing, China) for 5 min, stained in diaminobenzidine (DAB) and hematoxylin for 5 min, and dehydrated in ethanol. Finally, dried tissues were observed and photographed using a microscope (Leica Microsystems, Wetzlar, Germany).

### Western blotting

NRK-52E cells were lysed in radioimmunoprecipitation assay (RIPA) lysis buffer (Beyotime, Suzhou, China) to extract the total protein. The protein was separated in sodium dodecyl sulfate-polyacrylamide gel electrophoresis (SDS-PAGE) and transferred onto nitrocellulose membranes (Sigma-Aldrich, St. Louis, USA). Then, the membranes were placed in 5% milk for 2 h, incubated with 1000 times diluted antibodies of Col IV (ab6566), α-smooth muscle actin (α-SMA, ab32575), p62 (ab109012), microtubule-associated protein 1A/1B-light chain 3 (LC3)-II/I (ab128025), Beclin 1 (ab62557), phosphatase and tensin homolog deleted on chromosome ten (PTEN, ab32199), phosphatidylinositol 3-kinase (PI3K, ab189403), phosphorylated (p)-PI3K (ab182651), total (t)-protein kinase B (t-AKT, ab8805), p-AKT (ab38449), p-mTOR (p-mTOR, ab109268), and t-mTOR (ab63552, Abcam, Cambridge, USA) at 4°C overnight; then anti-mouse immunoglobulin G (IgG) antibody (1:2000; ab150113, Abcam, Cambridge, USA) was added and the membranes were incubated for 1 h. The bands were analyzed by an imaging system (Bio-Rad, Hercules, USA) and ImageJ software (NIH Image, Bethesda, USA).

### Quantitative reverse transcription-polymerase chain reaction (qRT-PCR)

Total RNA was extracted from NRK-52E cells using Invitrogen Trizol Reagent (Invitrogen Life Technologies, Carlsbad, USA), and complementary DNA (cDNA) was synthesized using SuperScript^™^ VILO^™^ cDNA Synthesis Kit (Thermo Fisher Scientific, Waltham, USA). The fragments of miR-155-5p were amplified in PCR using the following primers: forward 5’-UUAAUGCAAUCGUCAUAGGCGU-3’, reverse 5’-CCGUAUCACGAUUUGCAUUACAUU-3’. The PCR reaction conditions were 94°C for 5 min; 35 cycles of 94°C for 30 s, 55°C for 20 s, and 72°C for 20 s; and finally 72°C for 8 min. The results were analyzed by the 2^-ΔΔCt^ method [19].

### Dual luciferase reporter gene assay

The binding sites of miR-155-5p and PTEN were predicted on microRNA.org (http://www.microrna.org/microrna/home.do). HEK293 cells were transfected with miR-155-5p mimic, miR-155-5p inhibitor, NC mimic, or NC inhibitor and PTEN 3’ untranslated region (UTR)-wild type (WT) or PTEN 3’ UTR-mutation (MUT) were cotransfected into HEK293 cells using DEAE-Dextran Transfection Kit (Beyotime, Suzhou, China). Dual luciferase activity was measured by the Dual-Luciferase^®^ Reporter Assay System protocol (Promega, Madison, USA).

### Statistical analysis

Data were analyzed using IBM SPSS Statistics for Windows, Version 19.0. (IBM Corp., Armonk, NY, USA) and presented as mean ± standard deviation (SD). All experimental data were compared using one-way analysis of variance (ANOVA). The results were statistically significant when *p* < 0.05.

## RESULTS

### DHM improved autophagy and alleviated RIF in DN rats

**FIGURE 1:**
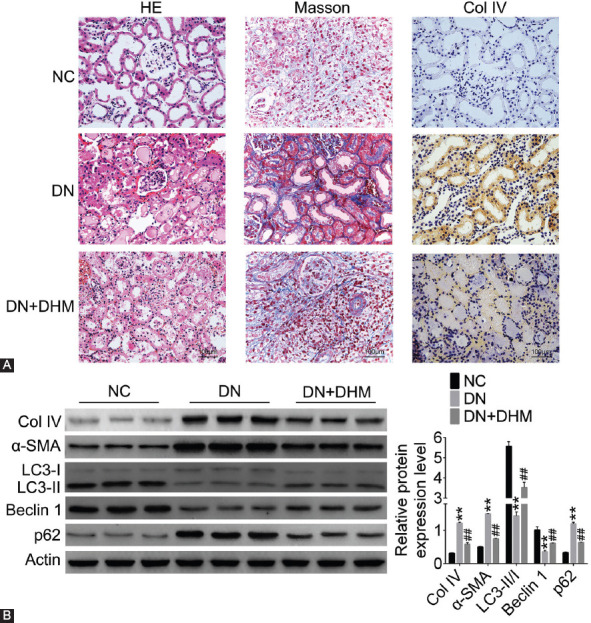
Dihydromyricetin (DHM) increased autophagy and reduced renal interstitial fibrosis (RIF) in diabetic nephropathy (DN) rats. Rats were treated with DHM at a dose of 100 mg/kg. (A) The degree of RIF was detected by hematoxylin-eosin (HE) staining, Masson’s trichrome staining, and immunohistochemistry (IHC) in DN rat model. (B) The fibrosis and autophagy-related proteins were detected by western blotting in DN rat model. **p < 0.01 compared with NC group; ^##^*p* < 0.01 compared with DN group. NC: Negative control; Col IV: Collagen-IV; α-SMA: α-smooth muscle actin; LC3: Microtubule-associated protein 1A/1B-light chain 3.

To investigate the effect of DHM on DN *in vivo*, we established a DN rat model to detect the degree of RIF. HE staining results indicated mild vacuolar degeneration of renal tubular epithelial cells, renal tubular dilatation, and renal interstitial macrophage infiltration in DN group (Figure 1A). DHM treatment improved DN-induced renal tubular epithelial degeneration and renal tubular dilatation. Masson staining results showed that the renal tissue morphology was normal, the renal interstitial collagen fibers were weakly expressed and blue, while the muscle fibers and red blood cells were red in NC group. However, in DN group, the renal tubules were obviously atrophied, the lumen was occluded, and the epithelial cells varied in size and arrangement. All these changes were mitigated in DN+DHM group (Figure 1A). IHC results showed that Col IV expression was upregulated in DN group compared to NC group, and that this upregulation was abolished in DN+DHM group (Figure 1A). Western blotting results (Figure 1B) showed that the protein expression level of Col IV, α-SMA, and p62 in DN group was higher than that in NC group. On the contrary, LC3-II/I and Beclin 1 expression level was downregulated in the renal tissue of DN rats. DHM treatment alleviated the upregulation of Col IV, α-SMA, and p62 and downregulation of LC3-II/I and Beclin 1 in DN+DHM group (*p* < 0.01).

### DHM promoted autophagy and alleviated fibrosis in HG-induced NRK-52E cells

**FIGURE 2:**
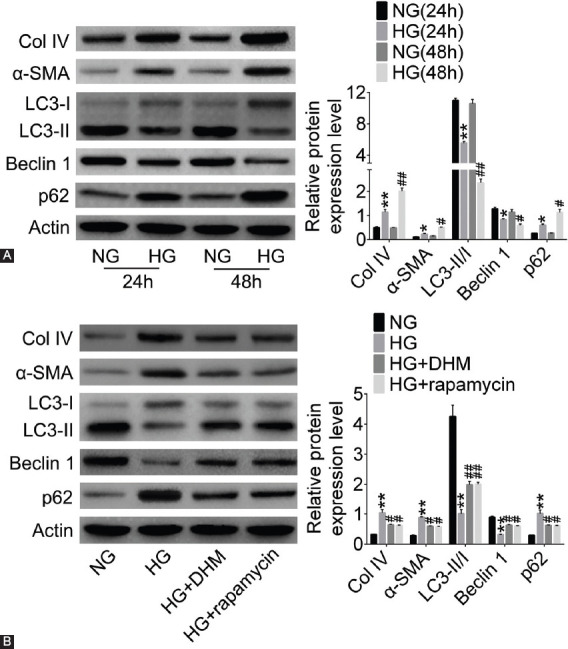
Dihydromyricetin (DHM) increased autophagy induced by high glucose (HG) and alleviated fibrosis in NRK-52E cells. The fibrosis and autophagy-related proteins were detected by western blotting in diabetic nephropathy (DN) cell model (A) at 24 h and 48 h and (B) in groups treated with DHM or rapamycin. *p < 0.05, **p < 0.01 compared with NG (24 h) or NG group; ^#^*p* < 0.05, ^##^*p* < 0.01 compared with NG (48 h) or HG group. NG: Normal glucose; Col IV: Collagen-IV; α-SMA: α-smooth muscle actin; LC3: Microtubule-associated protein 1A/1B-light chain 3.

We also established a cell model with NRK-52E cells to investigate the function of DHM in DN and detect its effect on cell autophagy and fibrosis. The protein expression level of Col IV, α-SMA, and p62 was upregulated, and LC3-II/I and Beclin 1 expression level was downregulated in HG group at 24 h and 48 h (*p* < 0.05 or *p* < 0.01; Figure 2A). The changes in protein expression at 24 h were greater than at 48 h. In addition, as shown in Figure 2B, the upregulation of Col IV, α-SMA, and p62 and downregulation of LC3-II/I and Beclin 1 induced by HG were reversed by both DHM and rapamycin treatments in NRK-52E cells (*p* < 0.05 or *p* < 0.01).

### DHM regulated the expression of miR-155-5p and PTEN

**FIGURE 3:**
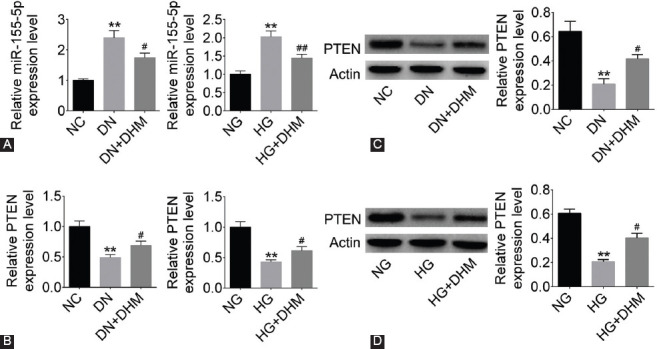
The expression of miR-155-5p was increased and that of phosphatase and tensin homolog deleted on chromosome ten (PTEN) was decreased in diabetic nephropathy (DN) groups, and dihydromyricetin (DHM) alleviated these changes. (A) MiR-155-5p expression was detected by quantitative reverse transcription-polymerase chain reaction (qRT-PCR) in DN rat and NRK-52E cell model. PTEN expression was detected by (B) qRT-PCR and (C and D) western blotting in DN rat and NRK-52E cell model. **p < 0.01 compared with NC or NG group; ^#^*p* < 0.05, ^##^*p* < 0.01 compared with DN or HG group. NC: Negative control; NG: Normal glucose; HG: High glucose.

To study the effect of DHM on miR-155-5p and PTEN *in vitro* and *in vivo*, we detected the mRNA and protein expression of miR-155-5p and PTEN by qRT-PCR or western blotting. The results showed that miR-155-5p expression was increased and PTEN expression was decreased in the renal tissues of rats in DN group and in NRK-52E cells in HG group (*p* < 0.01, Figure 3A and B). The upregulated expression of miR-155-5p and downregulated expression of PTEN were reversed by DHM treatment in rat tissues and NRK-52E cells (*p* < 0.05 or *p* < 0.01, Figure 3A and B). As shown in Figure 3C, PTEN expression level in DN group was lower than that in NC group, and DHM treatment inhibited DN-induced decrease in PTEN expression (*p* < 0.05 or *p* < 0.01). Similarly, in NRK-52E cells, PTEN expression level was downregulated in HG group, and in HG+DHM group DHM treatment attenuated the decreased expression of PTEN (*p* < 0.05 or *p* < 0.01, Figure 3D).

### PTEN was a target gene of miR-155-5p

**FIGURE 4:**
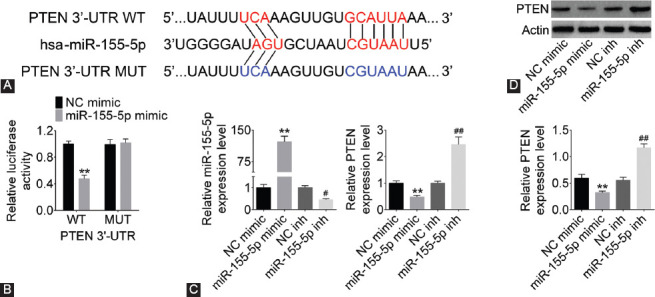
Phosphatase and tensin homolog deleted on chromosome ten (PTEN) was a target gene of miRNA-155-5p. (A) The binding sites were predicted on microRNA.org. (B) Dual luciferase activity was measured by dual luciferase reporter assay to confirm the relationship between PTEN and miRNA-155-5p. (C) MiR-155-5p and PTEN expression was detected by quantitative reverse transcription-polymerase chain reaction (qRT-PCR) in NRK-52E cells. (D) PTEN expression was detected by western blotting in NRK-52E cells. PTEN expression was upregulated with miRNA-155-5p suppression. **p < 0.01 compared with NC mimic group; ^#^*p* < 0.05, ^##^*p* < 0.01 compared with NC inhibitor group. WT: Wild type; UTR: Untranslated region; NC: Negative control; MUT: Mutant.

We used microRNA.org website to predict the target genes of miR-155-5p and found that there were six binding sites between miR-155-5p and PTEN (Figure 4A). Subsequently, we used dual luciferase reporter gene assay to verify the relationship between miR-155-5p and PTEN. As shown in Figure 4B, the relative luciferase activity was remarkably decreased in miR-155-5p mimic and PTEN-WT cotransfection group (*p* < 0.01) in NRK-52E cells, while cotransfection with PTEN-MUT had no effect on luciferase activity. The transfection efficiency of miR-155-5p mimic and miR-155-5p inhibitor is shown in Figure 4C (*p* < 0.05 or *p* < 0.01). PTEN expression level was decreased in miR-155-5p mimic group, while its expression was significantly increased in miR-155-5p inhibitor group (*p* < 0.01). Moreover, western blotting results showed that the protein expression of PTEN was downregulated in miR-155-5p mimic group and upregulated in miR-155-5p inhibitor group (*p* < 0.01, Figure 4D). These results suggested that PTEN was a direct target of miR-155-5p.

### DHM inhibited HG-induced fibrosis and promoted autophagy by inhibiting miR-155-5p expression in NRK-52E cells

**FIGURE 5:**
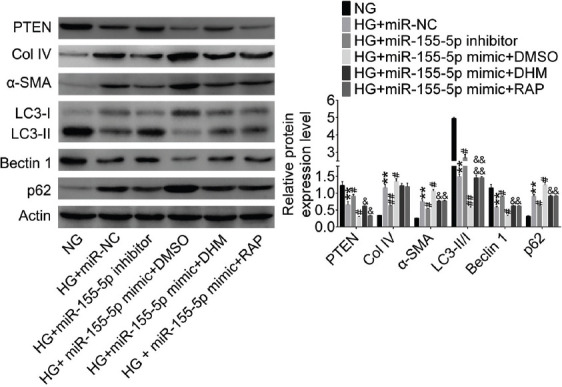
Dihydromyricetin (DHM) inhibited high glucose (HG)-induced fibrosis and promoted autophagy by inhibiting miR-155-5p expression in NRK-52E cells. PTEN, fibrosis-, and autophagy-related proteins were detected by western blotting in NRK-52E cells. **p < 0.01 compared with NG group; ^#^*p* < 0.05, ^##^*p* < 0.01 compared with HG+miR-NC group; ^&^*p* < 0.05, ^&&^*p* < 0.01 compared with HG+miR-155-5p DMSO group. NG: Normal glucose; HG: High glucose; NC: Negative control; DMSO: Dimethyl sulfoxide; Col IV: Collagen-IV; α-SMA: α-smooth muscle actin; LC3: Microtubule-associated protein 1A/1B-light chain 3; PTEN: Phosphatase and tensin homolog deleted on chromosome ten.

HG decreased the expression of PTEN, LC3-II/I, and Beclin 1 in NRK-52E cells and increased the expression of Col IV, α-SMA, and p62 (*p* < 0.01; Figure 5). MiR-155-5p knockdown decreased Col IV, α-SMA, and p62 expression and increased LC3-II/I and Beclin 1 expression in HG NRK-52E cells (*p* < 0.05). Conversely, miR-155-5p overexpression enhanced Col IV, α-SMA, and p62 expression and decreased LC3-II/I and Beclin 1 expression in HG group (*p* < 0.05). Furthermore, DHM and rapamycin both alleviated these changes induced by miR-155-5p overexpression and HG treatment in NRK-52E cells (*p* < 0.05).

### DHM promoted autophagy by inhibiting the PI3K/AKT/mTOR signaling pathway

**FIGURE 6:**
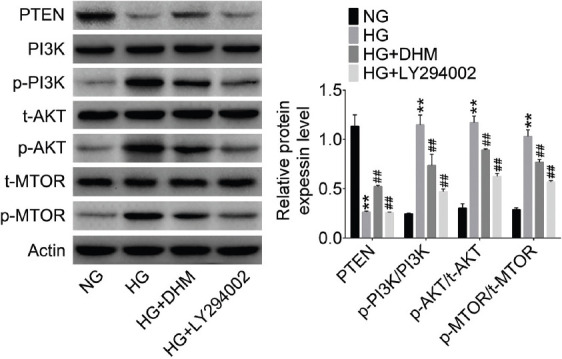
Dihydromyricetin (DHM) promoted autophagy by inhibiting phosphatidylinositol 3’-kinase/protein kinase B/mammalian target of rapamycin (PI3K/AKT/mTOR) signaling pathway. The pathway-related proteins were detected by western blotting in NRK-52E cells. **p < 0.01 compared with NG group; ^##^*p* < 0.01 compared with HG group. NG: Normal glucose; HG: High glucose; PTEN: Phosphatase and tensin homolog deleted on chromosome ten; p: Phosphorylated.

To further explore the effect of DHM on autophagy, we detected the expression of PI3K/AKT/mTOR signaling pathway-related proteins in NRK-52E cells by western blotting. As shown in Figure 6, the expression level of p-PI3K, p-AKT, and p-mTOR was significantly increased and the expression of PTEN was decreased in HG group compared with NG group (*p* < 0.01). DHM mitigated these HG-induced increases in p-PI3K, p-AKT, and p-mTOR and decrease in PTEN in HG+DHM group (*p* < 0.01). In addition, the expression levels of p-PI3K, p-AKT, and p-mTOR in HG+ LY294002 group were much lower than those in the HG group (*p* < 0.01). However, PI3K inhibitor (LY294002) had no effect on the expression of PTEN in HG-treated NRK-52E cells (*p* < 0.01).

## DISCUSSION

In this study, we established a DN rat model by intraperitoneal injection of STZ into SD rats. The HE and Masson staining results showed abnormal changes in the size of renal tubular, epithelial cells and the formation of collagen fibers in DN rat model. Interestingly, DHM treatment alleviated the above changes in the renal tubular, epithelial cells, indicating that DHM mitigated DN-induced RIF *in vivo*.

McClelland and Kantharidis suggested that miRNAs may serve as new biomarkers for the diagnosis of DN [20]. MiR-155-5p is mainly distributed in glomerular endothelial cells, mesangial cells, and renal tubular areas of the kidney. Moreover, miR-155-5p is reported to be highly expressed in kidney tissues of DN patients, and its expression level gradually increases with the progress of DN [21]. Our results showed that miR-155-5p expression was elevated in the tissues of rats in DN group and in NRK-52E cells in HG group, suggesting that miR-155-5p may be involved in the development of DN *in vivo* and *in vitro*. We also verified that PTEN was a target gene of miR-155-5p and its expression level was decreased in the DN rat and cell model. In addition, DHM could inhibit DN-induced changes of miR-155-5p and PTEN expression.

TGF-β1 in the kidney of DM rats stimulates the synthesis of Col I, Col III, Col IV, and Col V, which deposit in the glomeruli and promote basement membrane thickening, thereby promoting glomerulosclerosis and enhancing fibrosis [22]. α-SMA acts as an indicator of the degree of renal fibrosis. TGF-β1 expression is induced when α-SMA induces the conversion of resting renal fibroblasts into myofibroblasts [23-25]. Jiang et al. showed that DHM inhibits the expression of TGF-β1 and α-SMA by activating AMPK, reduces the synthesis of ECM and increases the decomposition, and slows down the occurrence and development of glomerulosclerosis and RIF [26]. We detected the expression level of Col IV and α-SMA in the DN rat and cell model. Our results demonstrated that DN induced RIF, while DHM treatment reduced DN-induced RIF development *in vivo* and *in vitro*. In addition, DHM protects heart function, reduces oxidative stress and the level of inflammatory factors, alleviates pathological changes, improves mitochondrial function, inhibits cardiomyocyte apoptosis, and restores autophagy in STZ-induced DN mice [27].

Because DN may be related to the inhibition of autophagy in renal cells and abnormal deposition of ECM [28], we also investigated the effect of DN on autophagy in the DN rat and cell model. We found that DN inhibited autophagy, while DHM treatment promoted autophagy *in vivo* and *in vitro*.

LC3II and p62/SQSTMI are the markers for autophagy [29-31]. The induction of autophagy is the main protective mechanism against cell damage and a possible target for improving renal damage associated with human kidney diseases [32]. The PI3K/AKT/mTOR signaling pathway is a classical regulatory pathway associated with cell autophagy [33]. PI3K is an upstream regulator of AKT activation. PI3K phosphorylates mTOR by activating AKT to regulate cell growth, survival, and hypertrophy [34]. A previous study reported that PTEN activation inhibits AKT/mTOR signaling in DN pathological injury [35,36]. In this study, miR-155-5p promoted RIF development and inhibited cell autophagy, while DHM inhibited RIF development and promoted cell autophagy by inhibiting miR-155-5p expression and by regulating the PI3K/AKT/mTOR signaling pathway in DN NRK-52E cells.

## CONCLUSION

This study demonstrated that DHM promotes autophagy and decreases the degree of RIF in DN by regulating miR-155-5p/PTEN signaling pathway. These findings provide new ideas for targeted therapy in DN and suggest a new theoretical framework for DHM treatment in DN. However, further studies are needed to clarify the underlying mechanism of DHM effects on DN development.
